# Improvement of Mitochondrial Activity and Fibrosis by Resveratrol Treatment in Mice with *Schistosoma japonicum* Infection

**DOI:** 10.3390/biom9110658

**Published:** 2019-10-25

**Authors:** Tina Tuwen Chen, Shihyi Peng, Yanjuan Wang, Yuan Hu, Yujuan Shen, Yuxin Xu, Jianhai Yin, Congshan Liu, Jianping Cao

**Affiliations:** 1 National Institute of Parasitic Diseases, Chinese Center for Disease Control and Prevention, Key Laboratory of Parasite and Vector Biology, MOH, Shanghai 200025, China; qooqoo0722@gmail.com (T.T.C.); wangyj@nipd.chinacdc.cn (Y.W.); 18930532676@163.com (Y.H.); amyshyj12@163.com (Y.S.); ipdxuyx@163.com (Y.X.); chart2543@163.com (J.Y.); lcs_hxy@hotmail.com (C.L.); 2 National Center for International Research on Tropical Diseases, Shanghai 200025, China; 3 WHO Collaborating Center for Tropical Diseases, Shanghai 200025, China; 4 Department of Biochemistry, School of Medicine, Tzu Chi University, Hualien 97004, Taiwan; pengsy@mail.tcu.edu.tw

**Keywords:** schistosomiasis, *Schistosoma japonicum* (*S. japonicum*), resveratrol (RSV), schistosomiasis-associated liver fibrosis (SSLF), mitochondrial function, *S. japonicum* adult worm

## Abstract

Schistosomiasis caused by *Schistosoma japonicum* is a major parasitic disease in the People’s Republic of China. Liver fibrosis is the main pathological mechanism of schistosomiasis, and it is also the major lesion. The common drug used for its treatment, praziquantel (PZQ), does not have a marked effect on liver fibrosis. Resveratrol (RSV), which is an antioxidant, improves mitochondrial function and also attenuates liver fibrosis. The combination of PZQ and RSV has been found to have a synergistic antischistosomal effect on *Schistosoma mansoni*; additionally, the activity of PZQ is enhanced in the presence of RSV. Here, we examine the therapeutic effects of RSV on the *S. japonicum* infection in a mouse model, and we investigate RSV as a novel therapeutic agent for mitochondrial function and schistosomiasis-associated liver fibrosis (SSLF). Mitochondrial membrane potential was examined using flow cytometry analysis. The expression of the mitochondrial biogenesis genes PGC-α and fibrosis-associated genes collagen I, collagen III and α-SMA were examined using western blot analysis. Fibrosis-associated histological changes were examined using Masson trichrome staining. Additionally, the effects of RSV on *S. japonicum* adult worms were examined using scanning electron microscopy and transmission electron microscopy. RSV treatment improved mitochondrial function by increasing membrane potential and increasing PGC-α expression (mitochondrial biogenesis). Further, RSV attenuated liver injury, including liver scarring, by decreasing collagen deposition and the extent of fibrosis, based on the decrease in expression of the fibrosis-related genes. RSV also decreased the adult worm count and caused considerable physical damage to the worm. These results indicate that RSV upregulates mitochondrial biogenesis and inhibits fibrosis. RSV may have potential as a therapeutic target for the treatment of fibrosis in schistosomiasis.

## 1. Introduction

Schistosomiasis is a water-borne parasitic disease that plagues many tropical and subtropical regions. It is a highly neglected tropical disease caused by parasitic helminth worms of the genus *Schistosoma*. The disease afflicts more than 230 million people in 76 countries, and 500–600 million people are at risk of infection [1]. (http://www.who.int/mediacentre/factsheets/fs115/en/index.html). The clinical features of schistosomiasis range from fever and headache to severe liver inflammation, liver scarring (collagen deposition and fibrosis) and organ failure [2,3,4].

The *S. japonicum* infection, which is one of the three major schistosomiasis conditions in humans, is still a public health issue in the People’s Republic of China [5]. Chronic infection with *S. japonicum* is characterized by hepatosplenic schistosomiasis, and the clinical symptoms include granuloma formation, periportal fibrosis, portal hypertension, hepatosplenomegaly, ascites and vascular shunt formation [2,6]. *S. japonicum* cercariae that are transmitted through the skin can lay a large number of eggs, which then pass through the sinusoidal endothelial vascular system and accumulate in the liver [7]. Maximal egg accumulation is observed at the site of the liver, and this is believed to play a major role in the pathogenesis of this disease [1]. The eggs secrete soluble egg antigens (SEAs), interact with various liver cells and elicit an egg-induced granulomatous inflammatory and general immune reaction, which leads to portal hypertension and, subsequently, schistosomiasis-associated liver fibrosis (SSLF) [8,9,10,11,12,13]. Although granuloma formation is induced to destroy the eggs and sequester or neutralise other pathogenic egg antigens, this process also leads to extensive fibrosis of host tissues. SSLF could develop into an irreversible advanced stage upon repeated exposure to the causative schistosome eggs. Despite recent progress in anti-schistosomal strategies, clinical management remains a challenge because SSLF is a complex, multi-step and often fatal disease [7].

Our previous study reported that the *S. mansoni* infection changes the mitochondrial morphology and promotes mitochondrial fragmentation in the mouse liver. In addition, it also affects mitochondrial function and attenuates mitochondrial membrane potential [14]. Furthermore, the *S. mansoni* infection was found to disrupt the mitochondrial dynamics of the mouse liver and induce mitochondrial fission [14]. Based on these results, it is possible that drugs that target mitochondrial morphology and function as well as the proteins involved in mitochondrial networks may present useful targets for the treatment of schistosomiasis.

Resveratrol (C_14_H_12_O_3_ or 3,5,4′-trihydroxystibene) is a natural non-flavonoid polyphenol found in abundance in fruits, vegetables, peanuts and red wine. Recent studies have reported that resveratrol (RSV) has diverse biochemical and physiological effects that are beneficial for one’s health [15], such as the anti-inflammatory, anti-oxidation, anti-fungal, antiviral, antibacterial and anti-parasitic effects of pure RSV or RSV combined with other compounds [16,17,18,19,20,21]. RSV has also been reported to attenuate and prevent liver fibrosis [22,23]. Importantly, it is well tolerated at high doses and does not have any side effects [24]. Previous studies indicate that mitochondria are a key downstream target of RSV, which can promote mitochondrial biogenesis and number [25,26]. In particular, RSV can regulate mitochondrial biogenesis and fission/fusion to attenuate cell damage induced by toxin treatment [26]. Several studies have shown that RSV improves mitochondrial function by activating peroxisomal proliferative activated receptor-γ coactivator 1α (PGC-1α) [27,28], the master regulator of mitochondrial biogenesis. PGC-1α enhances mitochondrial genes transcription, translation machinery [29,30,31] and elevates mitochondrial number and function [32,33,34,35]. Gouveia et al. demonstrated that the combination of PZQ and RSV has a moderate synergistic antischistosomal effect via a host-parasite model involving *Bioamphalaria glabrata* and the newly transformed schistosomula (NTS) of *S. mansoni* [36]. Therefore, we evaluated mitochondrial activity and fibrosis processes associated with schistosomiasis in the presence of RSV. Our findings indicate that RSV affects the mitochondrial mechanisms and fibrosis processes associated with the disease and provides a valuable approach for the treatment of schistosomiasis.

## 2. Materials and Methods

### 2.1. Ethical Statement

Animal care and all animal procedures were carried out in accordance with the Guidelines for the Care and Use of Laboratory Animals of the Shanghai Veterinary Research Institute. The study was approved by the Ethics Committee of the National Institute of Parasitic Diseases, Chinese Center for Disease Control and Prevention (approval ID: NIPD 2015–9).

### 2.2. Animals and Parasites

Male Balb/c mice (6–8 weeks old) were obtained from the Shanghai Laboratory Animal Center, Shanghai, China. *Oncomelania hupensis* (a snail species) harboring *S. japonicum* (SJ) cercariae were purchased from the Jiangsu Institute of Parasitic Diseases (Wuxi, China).

### 2.3. Establishment of Model Mice and RSV Treatment

The mice that were used to establish the *S. japonicum* infection model were percutaneously infected with 20 ± 2 *S. japonicum* cercariae via the shaved skin of the abdomen. Thirty mice were randomly assigned to the liver fibrosis (non-treatment) group. From this group, six mice were sacrificed at each of the following time points: 2, 3, 5, 8 and 10 weeks after infection. The remaining 30 mice were assigned to the RSV group. All 30 mice were treated with an RSV suspension (400 mg·kg^−1^·d^−1^) for 3 days by gastric gavage at 6 weeks after infection. Six mice were sacrificed at each of the following time points: 2, 3, 5, 8 and 10 weeks after infection.

### 2.4. Measurement of Mitochondrial Membrane Potential

Each liver sample was immersed in 10 mL phosphate-buffered saline that contained 5% fetal bovine serum (Gibco-BRL, Carlsbad, USA). Then we dispersed the cells from the liver by using a syringe needle. To recover these cells, we took them by centrifuging (250× *g*) at 4 °C for 5 min, and then re-suspended them in a red blood cell lysis buffer that contained 0.15 M ammonium chloride, 1 mM potassium bicarbonate and 0.1 mM disodium ethylenediamine tetraacetate (pH 7.2–7.4). Furthermore, we evaluated the mitochondrial transmembrane potential (Δφm) by JC-1 staining. Firstly, the cells from the mice liver were trypsinized and washed in phosphate-buffered saline (PBS). Then, we re-suspended these cells at 37 °C for 15 min in a culture medium that contained 10 g/mL JC-1 staining dye (Molecular Probes, Invitrogen, CA, USA). Subsequently, cells were washed twice with PBS and the pellet was re-suspended with PBS for flow cytometry analysis to detect the JC-1-stained cells (FACSalibur, BD Bioscience, San Jose, CA, USA).

### 2.5. Masson Staining

Liver specimens were fixed in 4% paraformaldehyde in PBS and dehydrated in a graded sucrose series. Then, Masson staining was performed using the standard methods (Solarbio, Beijing, China) to observe collagen fiber deposition. About five middle-power fields were randomly selected from each sample for analysis, and the ratio of the area occupied by collagen fibers to the total area was quantified using the Image Pro Plus 6.0 software (Media Cybernetics Inc., Rockville, MD, USA).

### 2.6. Western Blot Analysis

Liver tissues were homogenized in a RIPA lysis buffer (Beyotime, Shanghai, China) containing phenylmethanesulfonyl fluoride. After quantification, protein samples were boiled in 5× sodium dodecyl sulphate-polyacrylamide gel electrophoresis loading buffer for 10 min. Then, the samples were added into the wells of a 10% (*w*/*v*) polyacrylamide gel and transferred to a polyvinylidene fluoride membrane. The membranes were blocked with 10% milk at room temperature for 1 h and then incubated with antibodies against glyceral-dehyde-3-phosphate dehydrogenase (internal control; Proteintech, China), bactin (Proteintech, China), PGC-1α (Proteintech, China), collagen I (Proteintech, China), collagen III (Proteintech, China) and α-SMA (Proteintech, China) at 4 °C overnight. Then, the membranes were incubated with horseradish peroxidase-conjugated secondary antibodies at room temperature for 1 h. The membranes were examined using an enhanced chemiluminescence kit (Merck, Darmstadt, Germany).

### 2.7. Electron Microscope Analysis

For scanning electron microscope analysis, schistosomes were fixed with 2.5% glutaraldehyde in a Sorensen buffer (0.1 M, pH = 7.3) for 1 h and then washed in the same buffer. They were then fixed with 1% osmium tetroxide for 1 h. After fixation, the samples were dehydrated in a gradient ethanol series and dried to the critical point. The material was examined under a HITACHI S-4700 microscope with an accelerating voltage of 15 kV. For the transmission electron microscopic analysis, schistosomes were fixed with 2.5% glutaraldehyde in a Sorensen buffer (0.1 M, pH = 7.3) for 1 h and then washed in the same buffer. After fixation, they were post-fixed with 1% OsO_4_ in the same buffer and saccharose (0.25 M) for 2 h in the dark. Then, the schistosomes were dehydrated by incubation in successive aqueous ethanol solutions containing increasing percentages of ethanol (up to 100% (*v*/*v*)) before they were embedded in Spurr’s resin. Semi-thin sections (50 nm) were stained with 2% uranyl acetate in 50% ethanol and 8.5% lead citrate, and then examined on a HITACHI H-7500 electron microscope at an accelerating voltage of 80 kV.

### 2.8. Statistical Analysis

Data are expressed as the mean ± SD values. Comparison between two groups was performed with the Student unpaired *t*-test. The statistical significance of the data was analyzed with a *t*-test; *p* < 0.05 was considered to indicate statistical significance.

## 3. Results

### 3.1. RSV Treatment Affects the Expression of Genes Involved in Mitochondrial Biogenesis

**Figure 1:**
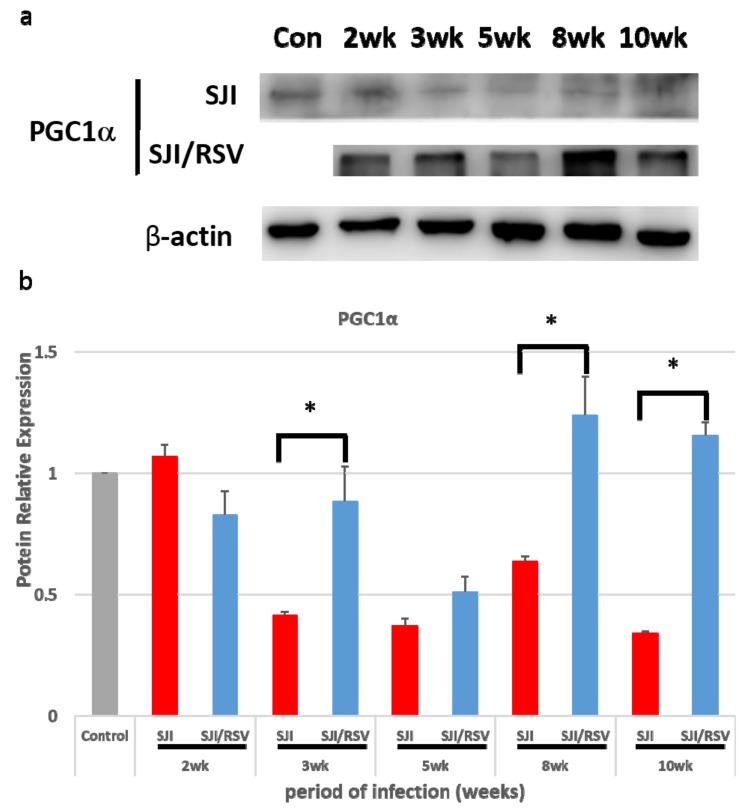
Resveratrol (RSV) increases peroxisomal proliferative activated receptor-γ coactivator 1α (PGC-1α) expression associated with mitochondrial biogenesis in the liver tissue of *S. japonicum*-infected mice. (**a**) Representative images of western blot gels for PGC-1α expression. (**b**) Quantitative changes measured by the ImageJ plugin software. RSV increased PGC-1α expression compared with the non-treated group (uninfected mice were used as the normal control group). Results are presented as mean ± SD (n = 5). * *p* < 0.05, unpaired Student *t* test.

In order to confirm whether the expression of genes involved in mitochondrial biogenesis is affected by RSV treatment of infected mice, PGC-1α expression was examined using western blot analysis. Compared to the non-treated infected mice, the PGC-1α protein showed higher expression in RSV-treated infected mice than in non-treated infected mice (*p* = 0.095588, 0.000662464, 0.105988197, 0.00857 and 0.00127 for the data obtained at -2, -3, -5, -8 and -10 weeks, respectively; Figure 1a,b).

### 3.2. RSV Improves Mitochondrial Function in S. japonicum-Infected Mice

**Figure 2:**
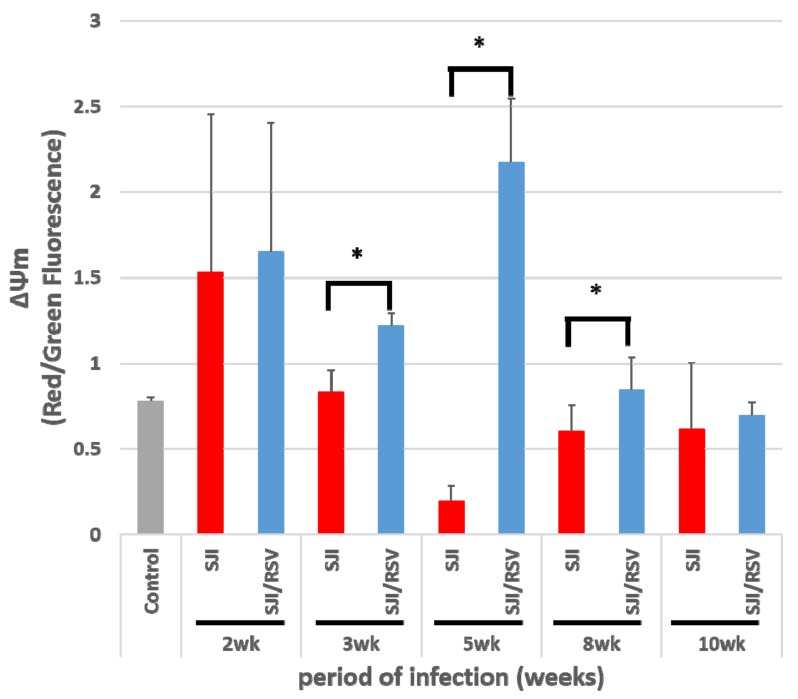
RSV improves the mitochondrial membrane potential (Δφm) of the liver in *S. japonicum*-infected mice. Representative data from flow cytometry analysis are presented. JC-1 staining of the mitochondria was used to assess the Δφm of mice liver. Δφm was higher in the RSV-treated group than in the non-treated group (uninfected mice were used as the normal control group). The values are presented as mean ± SD (n = 5). * *p* < 0.05, unpaired Student *t* test.

To determine whether the changes in mitochondrial function observed after RSV treatment in the infected mice was associated with increase in PGC-1α expression, mouse liver cells were stained with JC-1 to measure mitochondrial membrane potential (Δφm). FACS analysis revealed that the RSV-treated model mice showed a higher Δφm than the liver tissues of the untreated model mice (*p* = 0.435468, 0.00592, 0.00045, 0.07864 and 0.403903 for the data obtained at -2, -3, -5, -8 and -10 weeks, respectively, Figure 2). At 5 weeks after *S. japonicum* infection, a dramatic increase in Δφm was observed in the RSV-treated model mice compared to the non-treated model mice (*p* = 0.00045).

### 3.3. RSV Treatment Reduces SSLF

**Figure 3:**
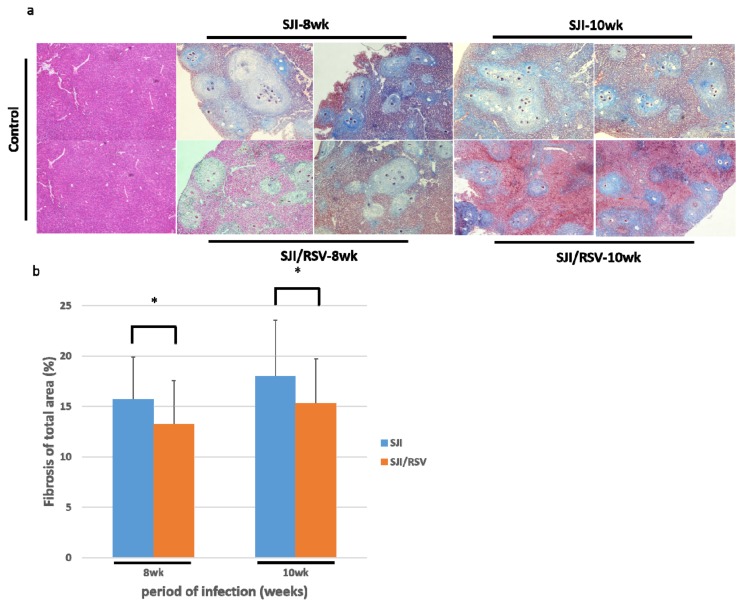
Pathological changes in liver tissues at 8 and 10 weeks after *S. japonicum* infection. (**a**) Representative images of Masson-stained liver tissue. Masson trichrome staining resulted in the collagen fiber being stained blue; cell nuclei, black; and background, red (original magnification, 100×). (**b**) The quantitative changes were measured with the ImageJ plugin software. RSV reduced collagen deposition compared to the non-treated mice (uninfected mice were used as the normal control group). Results are presented as mean ± SD (n = 5, with 50 fields per group). *p < 0.05, unpaired Student *t* test.

To study the effect of RSV on SSLF, liver samples were obtained from RSV-treated and non-treated mice that were euthanized at weeks 8 and 10 after infection. Masson trichrome staining of liver sections showed that the granulomatous areas and the severity of fibrosis were considerably reduced in the livers of RSV-treated mice as compared to the untreated mice (uninfected mice were used as the normal controls, ×100, Figure 3a).

The quantitative changes were measured using the ImageJ software (×100, *p* = 0.05584 and 0.01055, respectively, Figure 3b) (n = 5, 50 fields per group, unpaired Student *t*-test). Thus, RSV treatment may reduce *S. japonicum* egg-induced collagen deposition in the liver.

### 3.4. RSV Treatment Reduces Protein Expression of Fibrosis Markers

**Figure 4:**
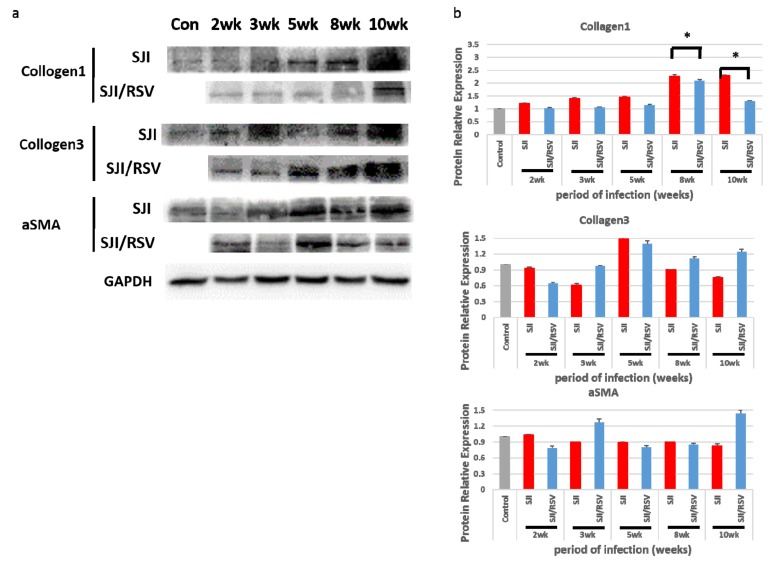
RSV decreases expression of fibrosis-related proteins in the liver tissue of *S. japonicum*-infected mice. (**a**) Representative images of western blot gels for collagen I, collagen III and α-SMA expression. (**b**) Quantitative changes measured with the ImageJ plugin software. RSV reduced the expression of all three genes compared to the non-treated mice (uninfected mice were used as the normal control group). Results are presented as mean ± SD (n = 5). * *p* < 0.05, unpaired Student *t* test.

Protein expression of collagen I, collagen III and α-SMA, which are commonly used markers of fibrosis, were examined using western blot analysis (Figure 4a). The quantitative changes were measured with the ImageJ software (uninfected mice were used as the normal control group, *p* < 0.05, Figure 4b). The data revealed that, with the exception of α-SMA expression (at 8 weeks), at both 8 weeks and 10 weeks, the expression of collagen I and collagen III were significantly higher in the liver of the non-treated mice compared to the RSV-treated mice (Figure 4b); this was consistent with the degree of SSLF indicated by Masson trichrome staining. Thus, RSV treatment may reduce SSLF.

### 3.5. RSV Treatment Reduces the S. japonicum Worm Count in Infected Mice

**Figure 5:**
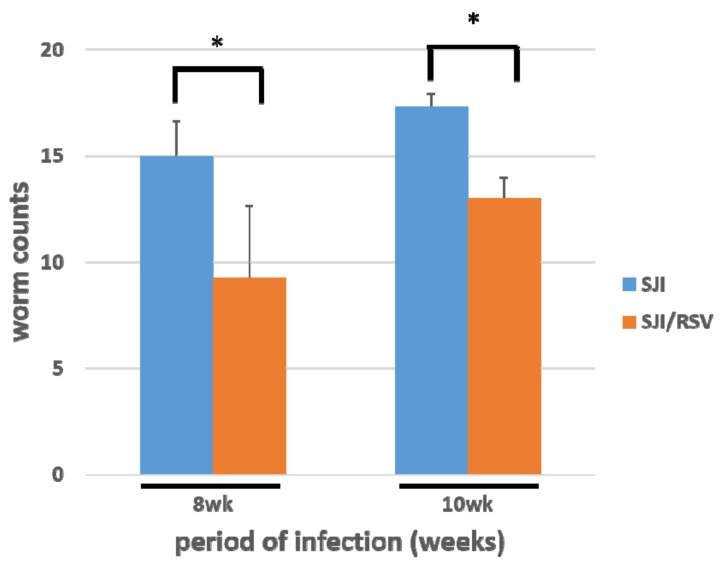
RSV reduces the count of *S. japonicum* worms obtained from mice at 8 and 10 weeks after infection. The worm count was significantly lower in the RSV-treated group than in the non-treated group at both 8 weeks and 10 weeks. Results are presented as mean ± SD (n = 10 for each group). *p < 0.05, unpaired Student *t* test.

Adult worms were obtained by portal perfusion and counted. The worm count was significantly lesser in the RSV-treated group than in the non-treated group (n = 10, *p* = 0.011307745 and 0.001445004, respectively, Figure 5).

### 3.6. RSV Treatment Causes Damage to S. japonicum Worms

**Figure 6:**
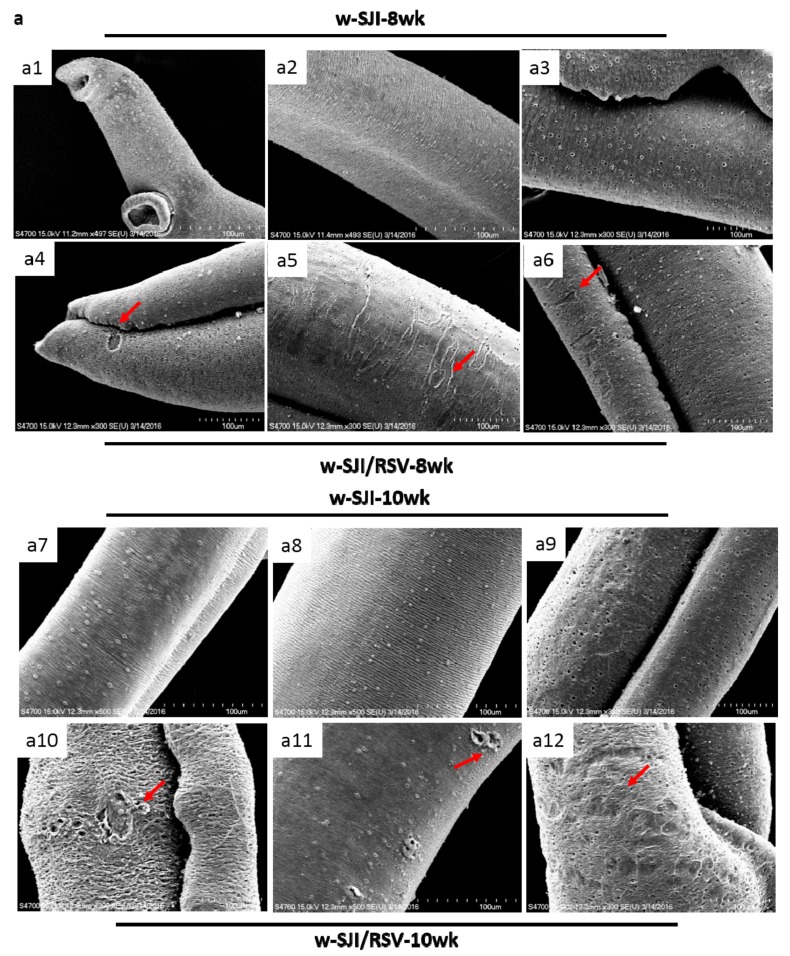

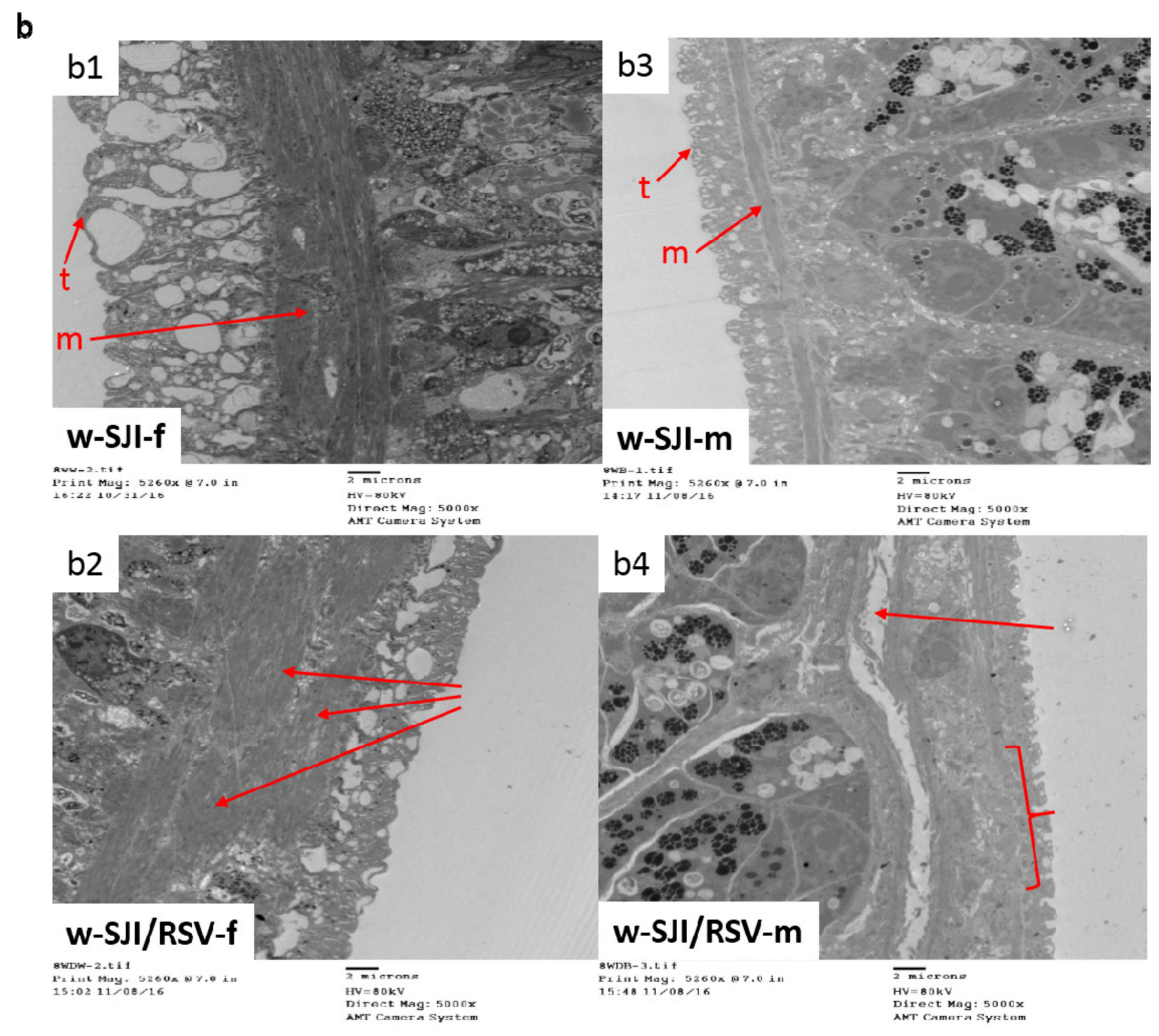
RSV damages the external epidermal tissue and internal cavity composition of adult *S. japonicum* worms obtained from infected mice at 8 and 10 weeks after infection. (**a**) Representative scanning electron microscopy images show the external epidermis of the adult worms at 8 and 10 weeks after infection. RSV not only induces swelling of the worms, but also induces loosening of the clefts and fusion of ridges. In addition, it results in the formation of holes and regions with extensive vesiculation, bursting of blebs, erosion of the tegument and sloughing (a4-6 and a10-12, red arrows). Non-treated mice were used as the control. (**b**) Representative transmission electron microscopy images showing that RSV not only damages the internal cavity of worms and results in thinning of the tegument, but also results in the swelling of muscle bundles (b2 and b4, red arrows). (n = 5 for each group).

The general morphology of adult worms obtained from the RSV-treated mice showed considerable damage when compared to the non-treated mice (Figure 6a). The tegument of control schistosomes (obtained from the non-treated group) exhibited normal circular ridges with regular clefts (Figure 6(a2,a8)). The transverse tegumental ridges encircling the body of the worm were less smooth on the surface of the worms in the RSV-treated group (Figure 6(a5,a11)). In addition, swelling in the RSV group induced the loosening of the clefts, and fusion of the ridges was also observed. The ridges were visibly damaged after treatment with RSV (Figure 6(a4–6,a10–a12)). RSV mainly induced swelling and the formation of holes and regions showing extensive vesiculation, bursting of blebs, erosion of the tegument and sloughing (Figure 6(a4–a6)). Another observation was erosion and peeling of the body of the worm after RSV treatment (Figure 6(a10–a12)). The blebs formed were so large that they fused together, inducing detachment of the larger parts of the tegument layer.

For ultramicrotomy analysis, ultrathin (50 nm) longitudinal sections of adult male and female *S. japonicum* worms were prepared. The following observations were made with transmission electron microscopy (10 sections were prepared for each worm). First, the tegumental matrix (t) and muscle bundles (m) were clearly visible on many transmission electron micrographs of the non-treated samples (Figure 6(b1,b3)). In the RSV-treated samples, the extent of tegumental damage significantly differed according to the parasite sections and sex of the worm. Second, in RSV-treated female worms, the tegumental matrix was obviously thinner than that in the control (non-treated) worms, but significant swelling of the underlying muscle bundles was observed (Figure 6(b2,b4)). In the case of RSV-treated males, a thinner tegumental matrix and swelling of the muscle bundles were clearly observed, and some of the underlying muscle bundles were found to disappear in Figure 6(b4), as depicted by the location of the curly bracket. In addition, there was an evident wound under the underlying muscle bundles (Figure 6(b4)).

### 3.7. RSV Causes Damage in S. japonicum Worms in a Dose-Dependent Manner

**Figure 7:**
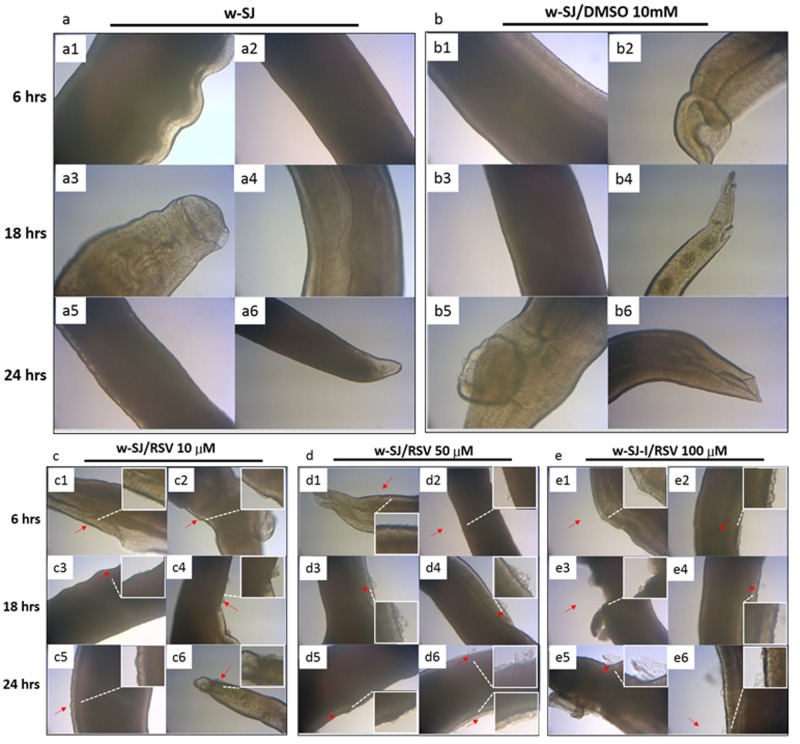
RSV causes damage to adult *S. japonicum* worms. Representative optical microscopy images show the external epidermal morphology of adult *S. japonicum* worms treated with RSV at a concentration of 10, 50 and 100 µM for 6, 18 and 24 h. The severity of body surface damage increased with increase in RSV concentration and treatment time (**c**–**e**, red arrows) compared with the positive controls (**a**) and negative controls (**b**). Drastic swelling and epidermal shedding were observed after treatment with RSV at 50 and 100 µM for 18 and 24 h (d3–d6 and e3–e6, red arrows). Uninfected worms were used as the positive controls, and worms treated with 10 mM DMSO were used as the negative controls (n = 5 for each group).

Adult SJ adult worms were treated in vitro with RSV at 10, 50 and 100 µM for 6, 18 and 24 h (Figure 7(c1–e6)); the untreated worms were used as positive controls (Figure 7(a1–a6)), and the worms treated with 10 mM DMSO were used as negative controls (Figure 7(b1–b6)).

The severity of body surface damage increased with increase in the RSV concentration and treatment time compared with the controls. In the RSV-treated group, the worms appeared convoluted and contracted, and they seemed stiff and exhibited drastic local swelling (Figure 7(c1–e6)). The swelling and epidermal shedding were pronounced after treatment with 50 and 100 µM RSV for 18 and 24 h (Figure 7(d3–d6,e3–e6)).

## 4. Discussion

Schistosomiasis is listed by WHO as the 17th most neglected tropical disease [37]. With the exception of malaria, schistosomiasis is the second most common human parasitic disease worldwide and it causes about 300,000 deaths every year. Among the three main human pathogenic schistosome species that are known to infect humans—*S. japonicum*, *S. mansoni* and *S. haematobium*—the most serious pathological lesions are caused by the *S. japonicum* infection. Until now, the mainstay treatment against schistosome-acquired infections has been chemotherapy. *S. japonicum* infection causes chronic, serious intestinal symptoms, including the well-known hepatosplenic schistosomiasis. Its serious progression is marked by the deposition of interstitial collagen types I and III, which together form fifibrotic tissue [38]. Unfortunately, until now, the exact pathological mechanisms of schistosomiasis-associated liver fibrosis are still unknown. After over 40 years of application, PZQ is still the only drug of choice for the treatment of schistosomiasis. PZQ is highly effective against all schistosome species in the adult stage, but it is not effective against immature parasites [39,40]. Liver fibrosis is the main pathological mechanism of schistosomiasis, and its complications are the major cause of death in cases of infection. However, the standard therapeutic dose of PZQ that is used for treatment does not markedly affect fibrosis.

In recent years, the search for new drugs in Chinese herbal medicine has become a new trend. In certain cases, anti-parasitic therapy, such as anti-toxoplasmosis treatment with a combination of traditional herbs, has been shown to slow down the replication of *T. gondii* and prolong the survival of mice [41]. In addition, anti-*Ichthyophthirius multifiliis* and anti-*Dactylogyrus ctenopharyngodonid* treatment with a combination of a Chinese medicine feed and ginger extract bath [42] and an anti-neosporosis treatment with Chinese medicine extracts (curcumin, artemether, etc.) have also had therapeutic effects [43]. As part of the immunological response to schistosomiasis, oxidative processes are triggered by the liberation of reactive oxygen species (ROS) and the disturbed cellular antioxidant homeostasis of the affected organs [44,45]. Additionally, we recently demonstrated that the *S. mansoni* infection increases the total ROS and NF-κB levels in mouse livers [46]. Therefore, antioxidant agents may have immense potential in the treatment of schistosomiasis.

RSV is a polyphenol found naturally in black grapes and red wine that has many biological activities, especially antioxidant activity [15]. RSV is completely non-toxic, is easily accessible due to its low market price, and has a well-defined target enzyme that is absent in mammals [47]. With regards to anti-parasite therapy, RSV may exert anti-*Trypanosoma cruzi* effects by inhibiting the activity of *T. cruzi* arginine kinase and exhibiting atrypanocidal activity [47]; anti-*Leishmania amazonensis* (both antipromastigote and antiamastigote) effects by increasing the percentage of promastigotes in the sub-G0/G1 phase of the cell cycle, reducing mitochondrial potential [48], increasing the number of annexin-V positive promastigotes [49] and inhibiting hypoxia inducible factor (HIF-1α) stimulation [50]; anti-*Raillietina echinobothrida* effects by altering the activity of certain energy metabolism-related enzymes in *R. echinobothrida* [51]; and anti-*Entamoeba histolytica* effects by inducing apoptosis-like death [52].

In the present study, we have evaluated the therapeutic effect of RSV in mice with the *S. japonicum* infection. RSV was found to improve *S. japonicum* infection-induced mitochondrial dysfunction and to alleviate the degree of fibrosis, as well as eventually being used to reduce the worm count and cause damage to the worm tissue.

In the present study, the *S. japonicum* infection was found to induce mitochondrial dysfunction and reduce mitochondrial biogenesis, as shown by the decrease in the mitochondrial membrane potential (Δφm) and the decrease in PGC-1α expression. Similarly, a recent study revealed that having the *S. mansoni* infection for 8 weeks induces mitochondria damage, and a decrease in the expression of genes involved in mitochondrial functional and biogenesis was noted [14]. In the present study, the inhibitory effects of *S. japonicum* on Δφm and PGC-1α expression were attenuated after 3 weeks of RSV treatment in the mice with *S. japonicum* infection. These findings are consistent with those of a previous study, which demonstrated that RSV alone or RSV combined with other compounds might have anti-parasitic effects [20,21,36].

The beneficial effects of RSV treatment have also been reported to improve liver fibrosis induced by CCL4 in mice [22,53] and in mice and adult humans with non-alcoholic fatty liver disease [54,55]. In particular, RSV treatment inhibits protein expression of the fibrosis markers α-SMA, COL-I, COL-IV and TGF-β [55]. Indeed, in the present study, at 3 weeks after RSV treatment, the protein expression of interstitial collagen types I and III and α-SMA was found to have increased. Additionally, at 8 and 10 weeks after RSV treatment, the extent of fibrosis was attenuated, as demonstrated by Masson staining. The main cause of fibrosis is periportal granulomatous inflammation around parasite eggs that is a result of the inflammatory response to the parasite [56]. In the present study, the number of worms in the RSV-treated *S. japonicum*-infected mice was obviously reduced at 8 and 10 weeks after RSV treatment. Additionally, the worms had also suffered considerable damage to their surface as well as interior as a result of the RSV treatment. In particular, in the in vitro experiments, the extent of epidermal erosion in the worms increased with higher RSV doses.

In the present study, RSV treatment for 8–10 weeks was found to decrease the extent of fibrosis. However, the degree of fibrosis did not reach normal levels. That is, even after RSV treatment, all the *S. japonicum*-infected mice still had severe SSLF; this was not concomitant with the increase in Δφm and PGC-1α protein expression caused by RSV. These data indicate that the effects of RSV on SSLF are not completely dependent on improvement of mitochondrial dysfunction.

The roles of mitochondrial dysfunction in inflammatory processes and cell death in fibrosis and liver injury have become increasingly evident [57]. Further, recent studies have demonstrated that RSV is important for preventing epithelial-to-mesenchymal transition and the subsequent tissue fibrosis via improvement of mitochondrial function (through an increase in Δφm and decrease in the expression of the fibrosis-associated α-SMA protein) [58]. RSV has also been found to attenuate cardiac injury in diabetic rats by ameliorating mitochondrial function and biogenesis via an increase in Δφm and PGC-1α expression [59]. Our results are consistent with reports in recent years that RSV not only increases mitochondrial function [60], but also protects mouse tissues against carbon tetrachloride, thioacetamide or nonalcoholic steatohepatitis-induced liver fibrosis [54,61,62,63]. That is, in the present study, we observed a significant increase in the levels of Δφm (with the exception of the 10-week RSV treatment) and the protein expression of PGC-1α (with the exception of the 5-week RSV treatment), and a reduction in the protein expression of collagen types I and III (with the exception of the 5-week RSV treatment) and α-SMA in *S. japonicum*-infected mice after RSV treatment for 3–10 weeks.

With regards to the effect of RSV treatment on *S. japonicum* worms, in the present study, we found that it caused damage to the external epidermal tissue and internal cavity composition of the worms. Further, the in vitro experiments demonstrated that RSV causes surface tissue stripping in the worm, and that the extent of injury and peeling off is dosage- and time-dependent. The results of our study show that RSV has in vitro as well as in vivo damaging effects on the *S. japonicum* worm; thus, RSV may be a promising candidate for future studies on schistosomiasis treatment. Previous studies have shown that RSV decreased the levels of the proinflammatory cytokine TNF-α in the supernatant of *Leishmania*-infected macrophages, but caused parasite death by increasing the percentage of promastigotes in the sub-G0/G1 phase of the cell cycle, reducing the mitochondrial potential and causing an elevated choline peak and CH2-to-CH3 ratio [48]. Similar to our findings, Ferreira et al. found that RSV had beneficial effects in the host (*Leishmania*-infected macrophages) but damaging effects on the parasite (promastigotes) [48].

In the present study, we observed that RSV causes an obvious reduction in the worm count and impairs both the surface and interior tissue of the worms in *S. japonicum*-infected mice; however, RSV does not seem to sufficiently affect the degree of fibrosis, with the result that SSLF is severe even after RSV treatment for 10 weeks. Thus, the results indicate that there might not be a direct causal relationship between improved mitochondrial function and alleviation of fibrosis.

Previously, we demonstrated that *S. mansoni* infection increases the total ROS and superoxide levels in mouse liver. RSV has been found to alleviate lung fibrosis induced by long-term particulate matter (PM) exposure through the inhibition of NLRP3 inflammasome activation and renal fibrosis in mice [64,65]. Since mitochondria are known to be the major sites of ROS production, it would be interesting to evaluate the effect of RSV on the ROS levels of *S. japonicum*-infected mice, and to determine whether the effect, if present, is concomitant with its effect on fibrosis. Furthermore, the effects of RSV on inflammatory processes are also worth further research in terms of the alleviation of mitochondrial dysfunction and fibrosis associated with schistosomiasis.

The present findings are highly interesting in the context of current treatments for schistosomiasis because PZQ, which is the mainstay treatment [66], plays a major role in killing adult worms but cannot protect the host tissue against SSLF [67]. Therefore, there is strong need for interventions to prevent or alleviate the development of SSLF, as it may improve the quality of life in the patients at later stages. In fact, previous studies have confirmed that SSLF can be reversed, and that the earlier SSLF treatment is started, the better the effect of the intervention is [27,68].

## 5. Conclusions

**Figure 8:**
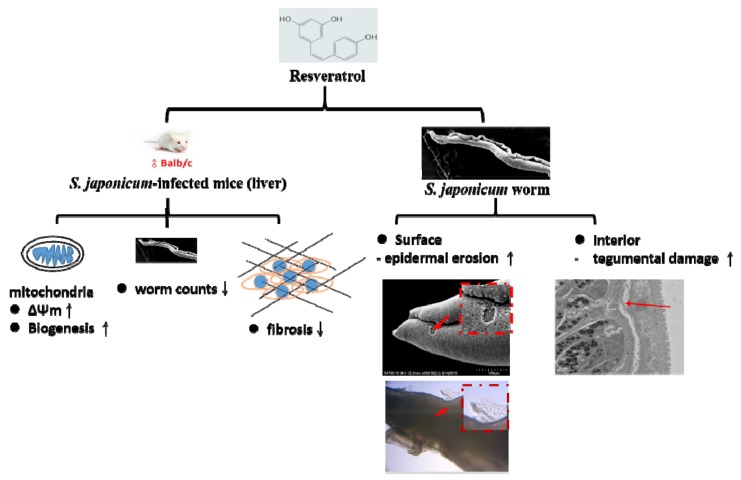
Schematic representation of the effects of RSV treatment on *S. japonicum*-infected mice. As shown in the schematic, in the liver of *S. japonicum*–infected mice, RSV results in an increase in Δφm and the protein expression of PGC-1α (which is associated with mitochondrial biogenesis), thus improving mitochondrial function. RSV treatment also results in a decrease in the extent of liver fibrosis, as indicated by a decrease in the protein expression of the fibrosis markers collagen I, collagen III and αSMA. As a final effect, RSV causes a decrease in the worm count in the liver and also causes damage to both the surface and interior tissues of the *S. japonicum* worms in *S. japonicum*-infected mice.

In summary, these results provide compelling evidence that RSV alleviates mitochondrial dysfunction and liver fibrosis caused by *S. japonicum* infection. The in vitro findings indicate that these effects of RSV were brought about through a decrease in the worm count and damage to the surface and interior tissue of the worms (Figure 8). Thus, RSV may have immense potential as a direct or indirect therapeutic agent for the treatment of schistosomiasis.
